# Cost-Effectiveness Analysis of Brief and Expanded Evidence-Based Risk Reduction Interventions for HIV-Infected People Who Inject Drugs in the United States

**DOI:** 10.1371/journal.pone.0116694

**Published:** 2015-02-06

**Authors:** Dahye L. Song, Frederick L. Altice, Michael M. Copenhaver, Elisa F. Long

**Affiliations:** 1 PhD Program in Health Policy, Harvard University, Cambridge, Massachusetts, United States of America; 2 AIDS Program, Section of Infectious Diseases, Yale University School of Medicine, New Haven, Connecticut, United States of America; 3 Division of Epidemiology of Microbial Diseases, Yale University School of Public Health, New Haven, Connecticut, United States of America; 4 Department of Allied Health Sciences, University of Connecticut, Storrs, Connecticut, United States of America; 5 UCLA Anderson School of Management, Los Angeles, California, United States of America; Örebro University, SWEDEN

## Abstract

**Aims:**

Two behavioral HIV prevention interventions for people who inject drugs (PWID) infected with HIV include the Holistic Health Recovery Program for HIV+ (HHRP+), a comprehensive evidence-based CDC-supported program, and an abbreviated Holistic Health for HIV (3H+) Program, an adapted HHRP+ version in treatment settings. We compared the projected health benefits and cost-effectiveness of both programs, in addition to opioid substitution therapy (OST), to the status quo in the U.S.

**Methods:**

A dynamic HIV transmission model calibrated to epidemic data of current US populations was created. Projected outcomes include future HIV incidence, HIV prevalence, and quality-adjusted life years (QALYs) gained under alternative strategies. Total medical costs were estimated to compare the cost-effectiveness of each strategy.

**Results:**

Over 10 years, expanding HHRP+ access to 80% of PWID could avert up to 29,000 HIV infections, or 6% of the projected total, at a cost of $7,777/QALY gained. Alternatively, 3H+ could avert 19,000 infections, but is slightly more cost-effective ($7,707/QALY), and remains so under widely varying effectiveness and cost assumptions. Nearly two-thirds of infections averted with either program are among non-PWIDs, due to reduced sexual transmission from PWID to their partners. Expanding these programs with broader OST coverage could avert up to 74,000 HIV infections over 10 years and reduce HIV prevalence from 16.5% to 14.1%, but is substantially more expensive than HHRP+ or 3H+ alone.

**Conclusions:**

Both behavioral interventions were effective and cost-effective at reducing HIV incidence among both PWID and the general adult population; however, 3H+, the economical HHRP+ version, was slightly more cost-effective than HHRP+.

## Introduction

Despite numerous evidence-based HIV prevention interventions, HIV incidence in the U.S. has remained unchanged over the past 15 years, with approximately 50,000 new infections occurring annually [1]. People who inject drugs (PWID), including male PWID who have sex with men (MSM), comprise nearly 20% of people living with HIV (PLHIV) and 11–13% of new infections [1–2]. PWID engage in increased injection-related and sexual risk behaviors that can transmit HIV to others, fueling HIV transmission to the general population [3].

Increased access to combination antiretroviral therapy (ART) markedly reduces HIV-related morbidity and mortality. Consistent ART access and optimal adherence suppresses viral replication, conferring benefits to uninfected populations by reducing sexual HIV transmission [4–5]. Additionally, several mathematical modeling analyses estimate substantial reductions in future HIV incidence with increased linkage to care and ART utilization [6–8]. Given concerns that the benefits of expanded ART might be offset by risk behavior disinhibition and the corresponding increase in sexually transmitted infections (STIs) that facilitate transmission, the role of evidence-based interventions (EBIs) for high-risk populations as part of a comprehensive HIV prevention and treatment approach has become exceedingly important [9].

EBIs that reduce needle-sharing, promote condom use, and improve ART adherence among PWID have demonstrated effectiveness [10], and a number of EBIs are widely available for PLHIV through the CDC's Diffusion of Effective Behavioral Interventions (DEBI) program [11]. Among these, the Holistic Health Recovery Program for HIV+s (HHRP+) serves as a `gold standard' among interventions targeting HIV-infected PWID [11]. HHRP+ is a comprehensive manual-guided risk reduction and health promotion intervention for HIV-infected PWID that centers on theory-based behavioral change [12]. Unlike most EBIs, HHRP+ potentially reduces HIV transmission by both improving ART adherence and by reducing sexual- and drug-related HIV risk behaviors [12–13].

Widespread implementation of EBIs has been constrained by limited resources necessary for proper implementation. A recent review comparing multiple-session EBIs with briefer interventions suggests that brief programs are likely to be more cost-effective and easier to implement [14]. Thus, an abbreviated Holistic Health for HIV (3H+) Program, an adapted HHRP+ version delivered in treatment settings, has been developed. The 3H+ is a theory driven, manual-guided, behavioral intervention that consists of four 45-minute weekly group meetings that are specifically designed to address sex- and drug-related transmission risk behavior and ART adherence among HIV-infected PWIDs [15]. A pilot study of 3H+ demonstrated significant improvement in both sexual- and drug-related risk measures [15]. To address this gap, a non-inferiority randomized controlled trial (RCT) comparing the briefer Holistic Health for HIV program (3H+) with HHRP+ is currently underway [15].

In the absence of findings from an ongoing randomized controlled trial (80 of 256 projected participants are enrolled), our goal is to estimate the projected health benefits and costs of implementing HHRP+ versus 3H+, through use of a mathematical epidemic model, at various levels of implementation, based on results from the original studies compared to treatment as usual. In the current HIV prevention and treatment era and in the absence of empirical data, we sought to model parameters that may markedly influence the outcomes of both expanded and abbreviated behavioral interventions. Modeling studies allow us to address the uncertainty of a number of outcome parameters in the short-term, yet allow for the eventual results of our proposed RCT to guide public health preventive recommendations. Further, our modeling study examines justification for the allocation of resources on briefer behavioral interventions from an economic perspective. Given the increasing constrained funding environment for HIV prevention efforts with a particularly challenging patient population, evaluating the cost-effectiveness of briefer interventions will provide support for their deployment of HIV prevention interventions for PWID until findings from the RCT become available. Thus, understanding the potential population-wide health gains and costs of secondary HIV prevention from these interventions through modeling will help translate observed risk behavior into meaningful, long-term health outcomes and help policymakers optimally allocate resources among competing HIV prevention programs.

## Materials and Methods

### Overview

We developed a customized HIV epidemic model to estimate the effectiveness and relative cost-effectiveness of two behavioral intervention programs (3H+ and HHRP+) for PWID in the U.S. The model used HIV transmission probabilities and contact rates among PWID and between PWID and the general adult population to project HIV incidence, prevalence, and HIV-related mortality over a 10-year horizon. Health benefits and costs were computed for each strategy considered. The model was calibrated to epidemic data of current U.S. populations and the natural history of HIV (Table 1). Published results from the original HHRP+ RCT and 3H+ pilot study were used for behavioral intervention parameters (Table 1) [12–13,15]. Sensitivity analyses were conducted on all parameters (S3 Table, S4 Table). The model was implemented in MATLAB (details available in S1 Appendix).

**Table 1:** Parameter Inputs for the Model and Uncertainty Ranges.

Parameter		Value	Range	Data sources
Population				
Initial adult population (age 15–64)		204,775,000		[31]
% PWID, 15–64 year olds		0.005	0.0013–0.03	[1,30,32–33]
Prevalence				
HIV prevalence PWID		0.150	0.087–0.243	[33–35]
HIV prevalence non-PWID		0.005	0.0017–0.0072	[1,31,36]
Initial disease stages for PLHIV				
Fraction with asymptotic HIV		0.65	0.55–0.75	[7,24–25]
Fraction with symptomatic HIV		0.05	0.05–0.15	[7,24–25]
Fraction with AIDS		0.30	0.15–0.35	[7,24–25]
Annual entry and exit rates				
Entry to population		0.025	0.01–0.05	[31,66]
Maturation		0.016	0.015–0.02	[31,66]
Non-AIDS death rate				
	PWID not on OST	0.0250	0.01–0.05	[25,27,37–38]
	PWID on OST	0.0125	0.01–0.025	[25,27,37–38]
	Non-PWID	0.0031	0.001–0.005	[31,67–68]
Annual HIV progression rates				
AIDS death rate				
	No ART	0.16	0.15–0.5	[10,24,69]
	ART	0.01	0.01–0.05	[10,24,69]
Progression rate				
	Asymptomatic to symptomatic	0.11	0.1–0.2	[24,27,45]
	Symptomatic to AIDS, no ART	0.33	0.2–0.5	[24,27,45]
	Symptomatic to AIDS, ART	0.055	0.05–0.1	[24,27,45]
ART				
Basecase access to ART				
	PWID	0.30	0.25–0.5	[17–18]
	Non-PWID	0.33	0.25–0.5	[17–18]
ART adherence				
	PWID	0.7	0.6–0.8	[17–18,70]
	PWID on OST or Program*	0.8	0.7–0.9	[17–18,70]
	Non-PWID	0.8	0.7–0.9	[17–18,70]
OST				
Proportion of PWID receiving OST		0.13	0.09–0.19	[16]
Injection behavior				
Average number of injections per year		150	100–300	[28,40]
Fraction of shared injections		0.2	0.1–0.4	[7,27,39–42]
Decrease in shared injections due to OST		0.75	0.5–0.99	[12–13];estimated
Additional decrease shared injections due to HHRP+		0.5	0.10–0.75	[12–13];estimated
Probability of transmission per shared injection				
	Asymptomatic HIV	0.001	0.0005–0.002	[7,24,27,45,54]
	Symptomatic HIV	0.001	0.0005–0.003	[7,24,27,45,54]
	AIDS	0.002	0.001–0.004	[7,24,27,45,54]
Reduction in injection infectivity due to ART		0.5	0.1–0.9	[7,24,27,45,54]
Sexual behavior				
Number of sexual partners per year				
	PWID	3.0	2.0–4.0	[43–45]
	Non-PWID	1.5	0.5–2.5	[43–45]
Fraction sexual contacts shared by PWID with PWID		0.4	0.2–0.6	[43–45]
Condom usage rate				
	PWID	0.25	0.1–0.4	[39,71]
	Non-PWID	0.20	0.1–0.4	[39,71]
	PWID in OST	0.40	0.2–0.5	[12–15,39,71];estimated
	PWID in HHRP+;3H+	0.5; 0.8	0.25–0.90	[12–15,39,71];estimated
Condom effectiveness		0.9	0.8–0.99	[72]
Chance of transmitting HIV per sexual partnership				
	Asymptomatic HIV	0.035	0.01–0.05	[7,25]
	Symptomatic HIV	0.04	0.01–0.05	[7,25]
	AIDS	0.08	0.03–0.1	[7,25]
	Decrease in transmission due to ART	0.9	0.5–0.99	[4,7,21–25]
Quality-of-life factor				
Non-PWID				
	No HIV	1.00	0.9–1.0	[7,73–74,79]
	Asymptomatic HIV	0.90	0.81–1.0	[7,73–74,79]
	Symptomatic HIV	0.78	0.7–0.86	[7,73–74,79]
	AIDS	0.70	0.63–0.77	[7,73–74,79]
Multiplicative factor, PWID not on OST		0.90	0.8–1.0	[7,24–25,27,52]
Multiplicative factor, PWID on OST		0.95	0.9–1.0	[7,24–25,27,52]
Multiplicative factor, on ART		1.10	1.0–1.3	[7,24–25,27,52,79]
Annual costs				
Discount rate		3%	0%-5%	[50]
Annual non-HIV-related healthcare costs		$7,578	$5,000–$9,000	[75]
Annual cost of ART		$15,300	$12,000–$18,000	[51]
Annual HIV-related healthcare costs				
	Asymptomatic HIV	$5,016	$3,000–$6,000	[76–77]
	Symptomatic HIV	$7,796	$5,000–$9,000	[76–77]
	Symptomatic HIV with ART	$7,045	$5,000–$8,000	[76–77]
	AIDS	$22,758	$15,000–$25,000	[76–77]
	AIDS with ART	$10,858	$6,000–$15,000	[76–77]
Annual cost of OST		$2,845	$1,500–$4,500	[13,19]
Annual cost of OST & HHRP+		$3,981	$2,000–$6,000	[13,19]
Annual cost of OST & 3H+		$3,081	$1,500–$4,500	[13,19]
Annual cost of HHRP+		$2,003	$1,000–$3,000	[13,19]
Annual cost of 3H+		$1,103	$500–$1,500	[13,19]

* Programs for PWID include either HHRP+ or 3H+.

PWID: people who inject drugs; HIV: human immunodeficiency virus; AIDS: acquired immune deficiency syndrome; ART: antiretroviral therapy; OST: opioid substitution therapy; HHRP+: Holistic Health Recovery Program for HIV+; 3H+: Holistic Health for HIV.

### Strategies Analyzed

We considered six different HIV prevention strategies for PWID and compared their relative effectiveness against the status quo. In the U.S., opioid substitution therapy (OST) using methadone or buprenorphine maintenance is currently available to only 13% of PWID [16]. Furthermore, only 30% of non-PWID and 33% of PWID are receiving ART [17–18]. These assumptions form the basis of our “status quo” scenario.

We first considered expanding either "HHRP+" or "3H+" to 80% of PWID. 3H+ is an adapted and shortened version of HHRP+ in treatment settings to improve the feasibility of implementation. Because HHRP+ is estimated to be twice as expensive as 3H+ [13,19], we also evaluated an "HHRP+ Low" strategy, which would reach 40% of PWID and cost approximately the same as 80% coverage of "3H+". In addition, we considered simultaneously expanding OST and a behavioral intervention ("OST/HHRP+", "OST/3H+"), since augmenting a behavioral program with OST was found to be effective [12–13]. For comparison, we modeled OST expansion alone ("OST"). Table 2 summarizes the coverage of each strategy.

**Table 2:** Base Case Assumptions and Estimated Number of HIV Infections Averted over 10 Years for Each Strategy.

	Status Quo*	OST	HHRP+	HHRP+ Low	3H+	OST & HHRP+	OST & 3H+
Fraction with access to harm reduction							
PWID	0.00	0.00	0.80	0.40	0.80	0.00	0.00
PWID who are on OST	0.00	0.00	0.80	0.40	0.80	0.80	0.80
Fraction of PWID with OST access	0.13	0.80	0.13	0.13	0.13	0.80	0.80
HIV prevalence after 10 years							
Overall (%)	0.57	0.56	0.56	0.57	0.57	0.56	0.56
Among PWID (%)	16.52	14.26	15.84	15.96	16.16	14.14	14.23
Among non-PWID (%)	0.51	0.50	0.50	0.50	0.50	0.50	0.50
HIV infections averted over 10 yr							
Total	-	60,986	28,862	21,991	19,229	74,426	70,547
Among PWID	-	24,926	10,949	8,593	7,143	28,895	27,807
Among non-PWID	-	36,060	17,913	13,398	12,086	45,531	42,741
Total receiving ART	552,650	599,110	545,870	547,960	546,210	568,910	569,280
Incremental Costs (US$ billions)	-	55.5	60.7	56.8	57.8	92.4	92.0
Incremental QALYs (millions)	-	2.3	7.8	6.8	7.5	8.7	8.5
ICER ($/QALY)	-	24,072	7,777	8,299	7,707	Dominated	Dominated

* Under the status quo, the total cost is 40,439 (US$ billions) and the total QALYs is 5270 (millions). For the other strategies, incremental costs and QALYs are relative to the status quo.

HIV: human immunodeficiency virus; PWID: people who inject drugs; OST: opioid substitution therapy; ART: antiretroviral therapy; QALY: quality-adjusted life year; ICER: incremental cost-effectiveness ratio relative to status quo.

### Antiretroviral Therapy and Opioid Substitution Therapy

The most recent (2013) U.S. guidelines recommend that all patients with chronic HIV infection be considered for ART initiation regardless of CD4 count [20–21]. After considering delays in early ART roll-out and uncertainty in the long-term health benefits of initiating ART at high CD4 counts, our model assumed that most patients initiate ART at a CD4 count of 350 cells/μL or when diagnosed with AIDS (CD4 count <200 cells/μL). We assumed that 70% of PWID and 80% of non-PWID receiving ART had sufficient adherence to achieve viral suppression [18].

With high levels of adherence, ART suppresses viral replication to undetectable levels, and markedly reduces the likelihood of sexual transmission from infected individuals to their sexual partners [4–5,22]. We assumed that ART reduces sexual transmission by 90% with optimal adherence [7,23–25] and by 58% for all patients with non-monogamous relationships, suboptimal ART adherence, or the presence of STIs [5]. Studies have also demonstrated that ART use among PWID is associated with reduced HIV incidence [26], but the extent to which ART reduces injection infectivity is not clear; thus, we assumed a 50% reduction in the chance of HIV transmission per shared injection, similar to other modeling studies [7,25,27].

Studies and systematic reviews have demonstrated the efficacy of OST for treating opioid dependence and preventing HIV infection [28–29]. PWID on OST share injecting equipment less frequently, both from less drug injection and a greater awareness of risk reduction strategies, but the estimated risk reduction range varies widely [28]. We assumed that PWID participating in OST will decrease needle sharing by 75%, based on the previous RCT results of HHRP+ [12]. OST potentially alters the number of sexual partnerships [28], but we conservatively estimated no changes in sexual behavior stemming directly from OST. We assumed that limited OST slots are filled immediately when a participant is lost, and thus a fixed proportion of PWID are assigned to OST slots at any given time.

Recent trends have suggested a decrease in HIV incidence among PWID, potentially as a result of various factors such as expanded OST, needle/syringe exchange programs (NEP), or a shift from injectable to non-injectable opioids. Although the availability of these harm reduction efforts might gradually increase, for our analysis we assumed that their availability remained constant. For comparison, our "OST" strategy qualitatively reflects a scale-up of such harm reduction efforts.

### Behavioral Interventions

HHRP+ involves 12 two-hour weekly manual-guided group sessions with comprehensive HIV risk reduction content that addresses the medical, emotional, and spiritual needs of drug-involved PLHIV [12]. HHRP+ participants significantly reduce self-reported and objective sexual- and drug-related behaviors and increase ART adherence compared to an enhanced Methadone Maintenance Program (MMP) alone [12–13]. These intervention benefits are reflected in our model by varying the number of risky injections and condom use (Table 1).

The 3H+ participants receive standard MMP and four 45-minute abbreviated sessions adapted from HHRP+. A pilot study of 3H+ demonstrated reduced drug- and sex-related HIV risk behaviors, and improved self-reported medical adherence skills [15]. We therefore assumed that 3H+ will have similar effects as HHRP+, but to an equal or lesser degree [15]. For the base-case analysis, we assumed that 3H+ effectiveness falls between no treatment and HHRP+ as specified in Table 1, but we varied this assumption in sensitivity analysis. (Table 1, S3 Table, S4 Table)

### Population

Our dynamic model divides the population into 22 compartments to account for variations in behaviors and disease status. The population is segmented by risk group: PWID, PWID on OST, and non-PWID. Each group is further stratified by their HIV infection status (uninfected, asymptomatic HIV, symptomatic HIV, or AIDS) and ART treatment status for those who are eligible (on ART, or not on ART). A schematic diagram and additional details are provided in S1 Fig.

We considered the adult population in the US in 2010 aged 15 to 64 years, which accounts for 96% of new HIV infections [1,31]. Approximately 0.5% of adults were PWID or PWID/MSM [1,30–32]. Initial HIV prevalence was estimated to be 15% among PWID [33–35] and 0.5% among non-PWID [1,31,36].

All individuals enter an uninfected state as either a PWID, PWID on OST, or non-PWID based on the national distribution of PWID. Individuals may exit from any compartment due to maturation when they reach 64 years, non-AIDS-related death, or AIDS-related death. The non-AIDS-related death rate is higher for PWID, due to excess mortality associated with opioid use [37–38], and OST was assumed to decrease the death rate by 50% [25,27]. The parameters used for analysis were determined from published sources (Table 1, S1 Table).

### Disease Transmission and Progression

In our model, HIV transmission occurs via shared injections between PWID, or via sexual contact between an uninfected and an infected individual. The overall transmission rate via needle-sharing was based on (1) the transmission probability per shared injection, (2) the likelihood of selecting an HIV-infected needle-sharing partner, and (3) the needle-sharing frequency among PWID, estimated from North American studies on injection behavior [39–42].

Similarly, the HIV transmission rate via sexual contact depended on the number of sexual partnerships between uninfected and infected individuals, as well as the chance of transmission per partnership. We allowed for preferential mixing between PWID and non-PWID, since PWID are more likely to select a PWID as a partner [43–44].

Once infected, the disease status of a patient can progress from asymptomatic to symptomatic HIV to AIDS. Without ART, the disease progression rates were estimated by natural-history data [24,45]. We assumed that the rate of disease progression is the same for PWID, PWID on OST, and non-PWID, and that ART slows disease progression from symptomatic HIV to AIDS by 80% [23,46–49].

### Model Validation

We validated our model externally using available U.S. demographic and epidemiological data from 2007 to 2010, adjusting only for initial adult populations, and lower levels of treatment access; the model closely approximated the total adult population, HIV prevalence, total HIV incidence, and HIV incidence among PWID (S2 Fig.).

### Health Outcomes and Costs

Population-level health benefits over 10 years, measured in quality-adjusted life years (QALYs), were calculated by summing the time spent in each health state and adjusting by a quality-of-life factor with lower values associated with more severe HIV status. Patients on ART had increased quality than those not receiving any treatment (Table 1). We also included future (discounted) QALYs accrued for all individuals alive at the end of the time horizon to include QALYs accrued at the end of the program up to death assuming individual remained in the same status.

Total costs included the direct costs of each intervention over the time horizon, as well as current and future healthcare costs throughout lifetime for the entire population. All costs and QALYs were discounted to the present time at 3% annually, following recommendations from the World Health Organization (WHO) guide to cost-effectiveness analyses [50]. Following standard cost-effectiveness analyses for health-related interventions, we considered all health care costs, including costs of HIV care and other health care, as well as the cost of methadone maintenance. The costs for providing the assessed interventions include labor, consumables such as medical supplies and medications. We did not take into account the capital costs such as building space and overhead costs, since the intervention is relatively short and does not require purchasing new medical devices or building a special facility. The annual cost for branded ART was estimated as $15,300 [51] and the costs of OST and HHRP+ were estimated from the available literature [13,52]. The 3H+, OST/HHRP+, and OST/3H+ strategies were assumed to have the same baseline costs (e.g., urine screens, medical care, case management, etc.) and information on unit cost for each component is provided elsewhere [13]. Each strategy was assumed to have different costs for the group sessions, however, due to differential time requirements. A detailed breakdown of costs is summarized in S2 Table. All costs were adjusted to 2012 US dollars according to the Medical Care component of the Consumer Price Index [19].

Finally, we computed the incremental cost-effectiveness ratio (ICER) of each strategy relative to the status quo, so that the cost-effectiveness of each program could be compared.

## Results

### HIV Infections Prevented

**Fig 1:**
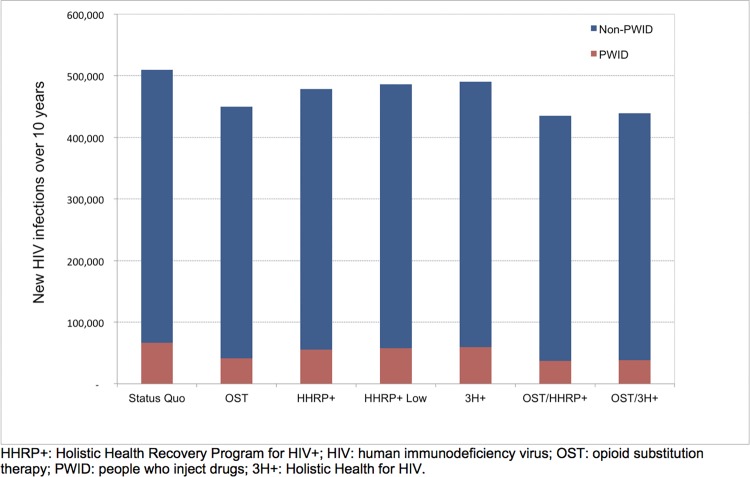
Estimated Number of New HIV Infections over 10 Years among PWID and Non-PWID for Each Strategy. HHRP+: Holistic Health Recovery Program for HIV+; HIV: human immunodeficiency virus; OST: opioid substitution therapy; PWID: people who inject drugs; 3H+: Holistic Health for HIV.

Under the status quo, we projected that 512,514 new infections (66,284 among PWID and 446,230 among non-PWID) would occur over 10 years (Fig. 1). With 80% HHRP+ coverage, 28,862 infections were averted, including 17,913 among non-PWID. Although the interventions targeted PWID only, this substantial benefit accrued to the general population because of reductions in risky sexual behavior by PWID, and due to reduced secondary transmission (i.e., preventing one PWID from acquiring HIV also eliminates the risk of infection to his/her future partners). Both 3H+ and “HHRP+ Low” strategies averted fewer infections, but with a similar fraction of infections prevented among non-PWID.

The combination OST/HHRP+ strategy averted the most infections (74,426), again with more than 60% of the infections prevented in non-PWID. OST/3H+ prevented slightly fewer infections (70,547) but with a similar proportion among non-PWID. Expanding OST by itself averted 60,986 total infections. The difference in infections averted between HHRP+ and 3H+ was reduced when combined with OST. The combination strategies prevented fewer infections than the sum of the individual programs because of diminishing returns (i.e., the same infection cannot be prevented twice).

### HIV Prevalence

**Fig 2:**
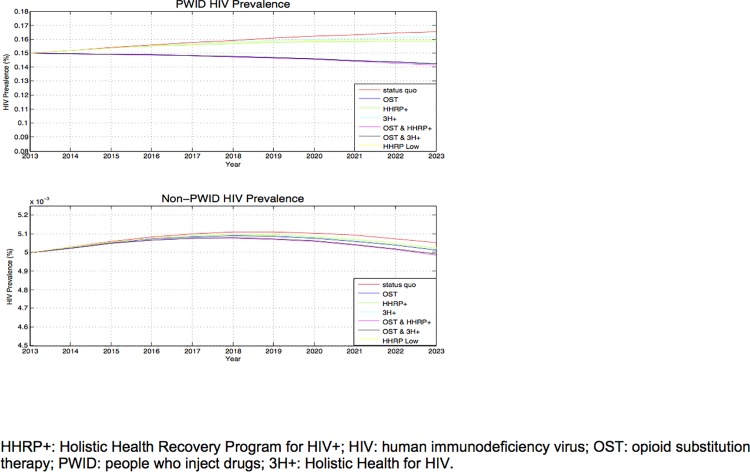
Estimated HIV Prevalence among PWID and Non-PWID. HHRP+: Holistic Health Recovery Program for HIV+; HIV: human immunodeficiency virus; OST: opioid substitution therapy; PWID: people who inject drugs; 3H+: Holistic Health for HIV.

Under the status quo, HIV prevalence among PWID was projected to reach 16.5% after 10 years (Fig. 2). Expanding OST could significantly reduce prevalence (to 14.3%), and HHRP+ and 3H+ also lowered prevalence (to 15.8% and 16.2%, respectively). Even with lower coverage levels, HHRP+ reduces prevalence to 16.0%. Combining HHRP+ or 3H+ with OST provided the greatest reduction in prevalence, reaching 14.1% and 14.2%, respectively.

Under the status quo and all strategies considered, HIV prevalence among non-PWID stayed almost constant at 0.5%. Although HIV incidence among non-PWID decreased, the reduction was not great enough to significantly affect the already low HIV prevalence among non-PWID.

### Cost-effectiveness Analysis

**Fig 3:**
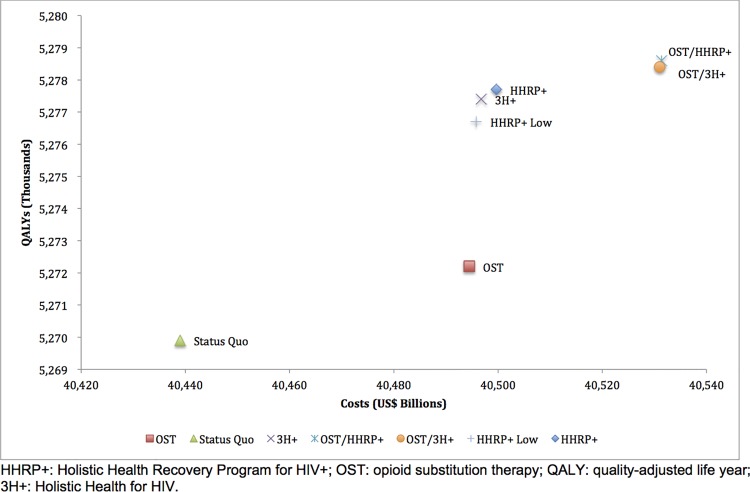
Cost-Effectiveness of Intervention Strategies. HHRP+: Holistic Health Recovery Program for HIV+; OST: opioid substitution therapy; QALY: quality-adjusted life year; 3H+: Holistic Health for HIV.

Either HHRP+ or 3H+ alone were the most cost-effective strategies, costing only $7,777 and $7,707 per QALY gained for HHRP+ and 3H+, respectively (Fig. 3). When comparing HHRP+ Low to 3H+, which have similar programmatic costs, 3H+ was more cost-effective than HHRP+. In other words, for a constrained budget, achieving 80% utilization of 3H+ is more cost-effective than 40% utilization of HHRP+, even if 3H+ is less effective per participant. All five strategies were highly cost-effective according to WHO's cost-effectiveness guidelines, costing less per QALY gained than the U.S. GDP per capita [53].

Among single strategies, expanding OST alone was the least cost-effective option ($24,072 per QALY gained). Combining OST with HHRP+ or 3H+ was extendedly dominated relative to implementing HHRP+ or 3H+ strategies alone because of lower ICER.

**Fig 4:**
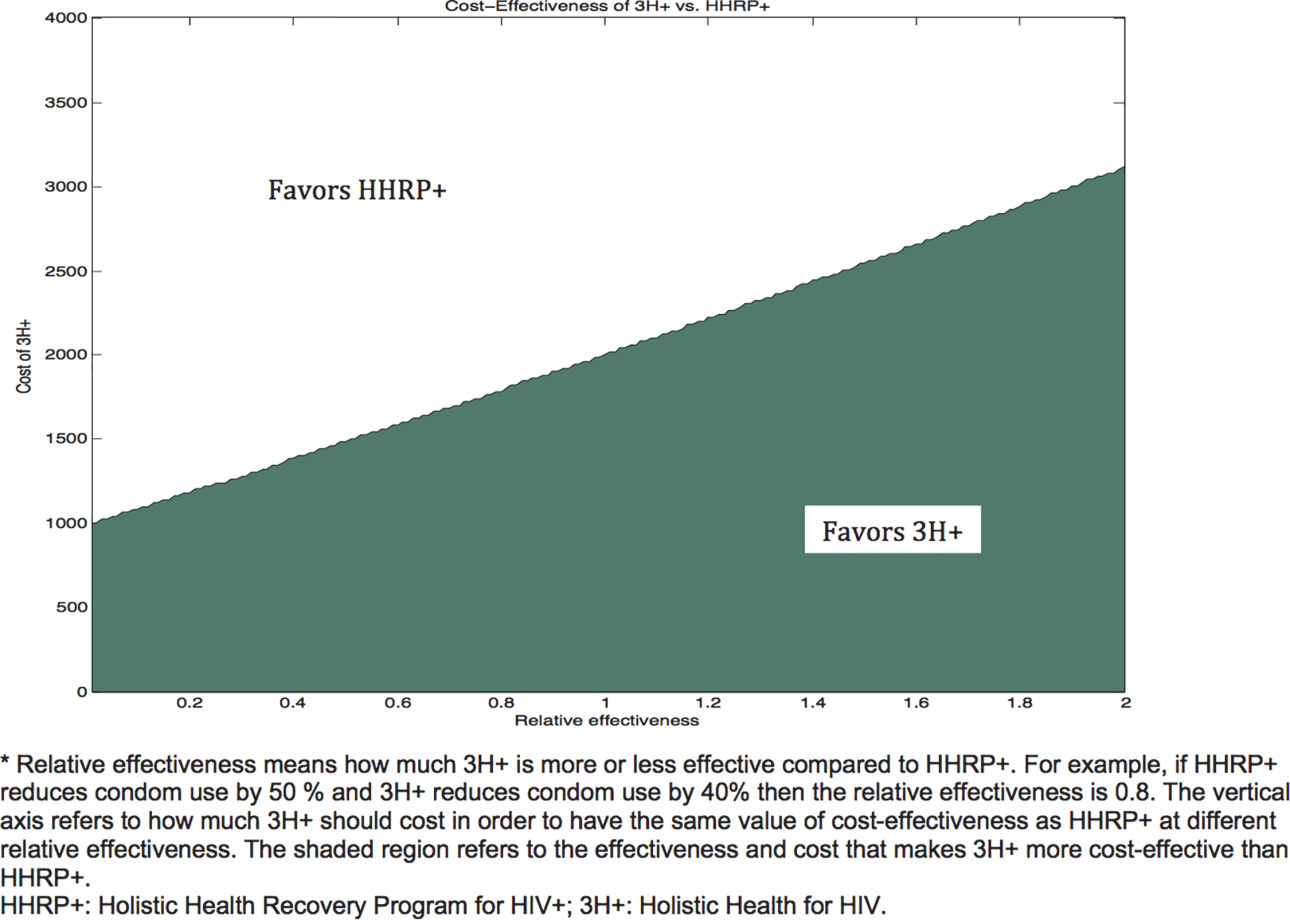
Relative Effectiveness vs. Cost of 3H+ vs. HHRP+. HHRP+: Holistic Health Recovery Program for HIV+; 3H+: Holistic Health for HIV.

We varied the effectiveness and the cost of 3H+ in comparison to HHRP+ to determine the threshold above which 3H+ becomes more cost-effective. We adjusted the behavioral parameters (risky injections and condom use) of 3H+ so the relative effectiveness varied from 0 (no effect) to 2 (twice the effectiveness of HHRP+), where 1 implies the same parameter values used for HHRP+. For each effectiveness ratio, the shaded region of Fig. 4 indicates the price range of the 3H+ intervention such that 3H+ is more cost-effective than HHRP+. The initial cost estimate of 3H+ was $1,103; therefore, 3H+ was preferred to HHRP+ if 3H+ was at least 6% as effective as HHRP+.

### Sensitivity Analyses

To address uncertainty in parameter estimates, we performed one-way sensitivity analyses by varying each parameter over its value range provided in Table 1 and calculating the range of infections averted (S3 Table). The parameter ranges are chosen based on the lowest and highest values estimated from literature and the sources are provided in Table 1. If complete ranges were not available from literature, we varied the range from 50% to the 150% of the value used, following standard sensitivity analysis conventions. We performed extensive sensitivity analyses on the following key parameters that were most sensitive: injection and sexual transmission infectivity; risky injection behaviors; risky sexual behaviors; and the effect of various behavioral interventions.

Because of limited data on the transmission probability per contaminated injection [54], we varied this parameter widely. Doubling the transmission probability resulted in 37,858 more infections averted for the HHRP+ strategy, and 23,440 averted for the 3H+ strategy. When infectivity was halved, HHRP+ and 3H+ became less cost-effective because overall transmission among PWID was greatly reduced, thus mitigating the benefits of either program.

The number of infections averted among non-PWID was sensitive to changes in sexual transmission probabilities. For the HHRP+ strategy, doubling the chance of transmission resulted in 2,464 additional infections averted among PWID and 6,564 among non-PWID. This highlights that reducing sexual transmission between PWID and non-PWID is a key determinant in controlling the epidemic for the overall population.

Prescription opioid abuse is growing in the U.S. [55] and evidence suggests that this is associated with transition to injection drug use [56–57]. Moreover, as heroin purity decreases, shifts toward increased injection and resultant risky injection practices occur among PWIDs [58–59]. To address this uncertainty, we varied risky injecting behavior by varying the average number of shared injections per PWID. Doubling the number of shared injections resulted in twice as many infections averted with each behavioral intervention. When the number of risky injections was very low (15 per person-year), both behavioral interventions were much less cost-effective. Variations in the number of sexual partnerships had a greater impact on the number of infections averted among non-PWID than among PWID, because sexual contact causes the majority of new infections in non-PWID. Variations in condom use and ART effectiveness at reducing injection and sexual infectivity did not change the relative ranking of each intervention.

In general, HHRP+ and 3H+ were more cost-effective than OST in all sensitivity analyses, and 3H+ was always more cost-effective than HHRP+. Finally, we varied the cost of OST to see if a lower cost would make the intervention more cost-effective than behavioral interventions. Even when we considered a $1,500 annual cost of OST, lower than the cost of HHRP+ ($2,003), OST was still the least cost-effective among all the individual strategies.

## Discussion

Our study used a tailored HIV epidemic model to examine the potential population-wide health gains and costs of HIV prevention through behavioral interventions (HHRP+ vs. 3H+) for HIV-infected PWID. Although prior studies found that psychological and behavioral interventions for reducing injection and sexual risk behavior are effective at preventing HIV in PWID [12,14,60–61], our study is the first to evaluate the broader epidemic impact and cost-effectiveness of a targeted behavioral intervention for HIV-infected PWID in the U.S.

We compared six different intervention strategies and found that both HHRP+ and 3H+ were effective at averting HIV infections and lowering HIV prevalence among PWIDs. Notably, these interventions were also very effective at averting infections among the general population, reducing projected HIV incidence among non-PWID by 10%. Thus, an effective behavioral intervention that broadly reaches HIV+ PWID could substantially bend the epidemic curve downward. Combining HHRP+ or 3H+ with OST averted more infections than expanding OST alone. These results support other findings that combination prevention programs are most effective [62]. We showed that HIV prevalence among PWID is expected to steadily increase without further interventions, but OST/HHRP+ or OST/3H+ would reverse this trend if widely implemented.

Our findings underscore how behavioral interventions for HIV-infected PWID can be both effective and cost-effective. Alone, these interventions prevented fewer infections than when combined with OST, yet both were more cost-effective. This is because the same degree of decrease in transmission risk behavior that can be accomplished via OST can be achieved with behavioral interventions at a lower cost. Furthermore, given a fixed budget, 3H+ is more cost-effective than HHRP+, across a range of parameter assumptions. The threshold for which 3H+ became more cost-effective than HHRP+ was very low: even if we assumed 3H+ is only half as effective as HHRP+ in preventing HIV transmission, it would remain the preferred approach as long as its price remained less than 75% that of HHRP+. Overall, investing in any of the five strategies could be highly economically efficient. The WHO cost-effectiveness guidelines define highly cost-effective interventions as ones that that cost less than the per capita gross domestic product (GDP) per QALY gained [53]. The U.S. GDP per capita was $53,143 in 2013 [78] and therefore, expansion of OST, behavioral interventions or combination of both are highly cost-effective and justify the comparative effectiveness study that is now underway.

Our results also demonstrate that even with 80% coverage among PWID, neither behavioral interventions nor OST alone significantly impact overall U.S. HIV prevalence, likely due to increased life expectancy for individuals on ART, and the relatively low HIV prevalence among the general population. In order to eliminate the U.S. HIV epidemic, reducing risk behaviors from other risk groups (e.g., MSM and high-risk heterosexuals) concurrently with PWID is crucial. Our assumptions are more conservative than other modeling studies that examined higher HIV prevalence settings and found more dramatic reductions from expanding OST [25,27,63]; however, these studies assumed lower ART access, and our study projected a smaller impact of OST on HIV prevalence due to higher ART use.

Our study has limitations that must be noted. First, the behavioral parameters were estimated from available RCTs, with some outcomes measuring patients' knowledge and motivation, which may not always translate into actual behavior change, although there is a documented correlation between these variables [64–65]. Sensitivity analyses examined this variation. Findings from the completed RCT comparing 3H+ with HHRP+ will refine these assumptions. Second, we assumed constant behavior and cost throughout the time horizon, assuming that the interventions’ effect is durable throughout the course of the patients' lifetime. Long-term outcomes from the RCT would refine our assumptions. The cost of ART, OST and the harm reduction programs might also decrease with the anticipated approval of generic drugs. Though we found through sensitivity analysis that varying the cost of OST did not change the results significantly, better estimates of HIV-related intervention costs would be helpful. Third, NEP coverage was not modeled, but in the absence of federal funding, it is not likely to change appreciably in the near future. Last, we did not take into account the productivity gains and losses due to interventions in the CEA, following the recommendation from WHO guide to cost-effectiveness analyses [50]. We conservatively assumed that the productivity loss of PWID due to any intervention is not significant, given the intermittent and short duration of interventions. Although productivity gains may arise from improved health as an outcome of the intervention, we did not include such gains in our analysis, since estimation of the amount is out of the scope of this analysis, and underestimates the maximal benefit afforded by either intervention. Consequently, inclusion of potential productivity gains in the analysis would likely shift the ICER of the interventions to be even more favorable compared to the status quo.

For generalizability, we used the average data representative of the U.S., and our sensitivity analyses indicate that the general pattern of our findings remained the same under variation of the HIV prevalence among PWIDs. It is important to note, however, that the epidemiology of HIV and AIDS in the United States is not evenly distributed across regions. We also expect that there would be some variation in costs of the program by location, and the benefits of the treatment may also vary depending on the characteristics of the PWID receiving the various interventions. Thus, generalization of our results to any specific regions or settings should be made with caution after assessing the local drug use context.

In conclusion, these findings suggest that behavioral intervention programs targeting HIV-infected PWID (HHRP+ or 3H+) are both effective and cost-effective at reducing HIV incidence among both PWID and the general adult US population. It is a commonly addressed concern that feasibility of implementing behavioral interventions among PWID is challenging, as these are a marginalized and stigmatized group who are challenging to engage, least able to access and arguably less likely to utilize HIV prevention, care, and treatment services in the absence of supportive services. Consequently, this supports the need for developing and tailoring HIV prevention programs to effectively target, reach, and address the particular needs of PWID. If 3H+ is confirmed to show comparable effectiveness to HHRP+ based on the ongoing RCT, our study supports the potential population-wide benefits of 3H+ as a more cost-effective alternative to HHRP+ and OST.

## Supporting Information

S1 Fig Simplified Schematic Diagram of Dynamic Compartmental Model. PWID: people who inject drugs; HIV: human immunodeficiency virus; AIDS: acquired immune deficiency syndrome; ART: antiretroviral therapy; OST: opioid substitution therapy; HHRP+: Holistic Health Recovery Program for HIV+; 3H+: Holistic Health for HIV.
(TIFF)

S2 Fig Results from Model Calibration. PWID: people who inject drugs; HIV: human immunodeficiency virus; AIDS: acquired immune deficiency syndrome.
(TIFF)

S1 Table Summary of Notations. HIV: human immunodeficiency virus; AIDS: acquired immune deficiency syndrome; ART: antiretroviral therapy; OST: opioid substitution therapy; HHRP+: Holistic Health Recovery Program for HIV+; 3H+: Holistic Health for HIV.
(TIFF)

S2 Table Utilization and Cost of Services per Person. OST: opioid substitution therapy; HHRP+: Holistic Health Recovery Program for HIV+; 3H+: Holistic Health for HIV.
(TIFF)

S3 Table Changes in Infections Averted of the "HHRP" Strategy Compared to the Status Quo for Each Parameter. PWID: people who inject drugs; HIV: human immunodeficiency virus; AIDS: acquired immune deficiency syndrome; ART: antiretroviral therapy; OST: opioid substitution therapy; HHRP+: Holistic Health Recovery Program for HIV+; 3H+: Holistic Health for HIV.
(TIFF)

S4 Table Results of Sensitivity Analysis on Key Parameters. PWID: people who inject drugs; HIV: human immunodeficiency virus; AIDS: acquired immune deficiency syndrome; ART: antiretroviral therapy; OST: opioid substitution therapy; HHRP+: Holistic Health Recovery Program for HIV+; 3H+: Holistic Health for HIV.
(TIFF)

S1 Appendix Technical Appendix. (TIFF)
