# Supplementary figures and images for: Cost-Effectiveness Analysis of Brief and Expanded Evidence-Based Risk Reduction Interventions for HIV-Infected People Who Inject Drugs in the United States
Source: PLoS One. 2015 Feb 6;10(2):e0116694. doi: 10.1371/journal.pone.0116694 (PMC4320073; doi:10.1371/journal.pone.0116694)

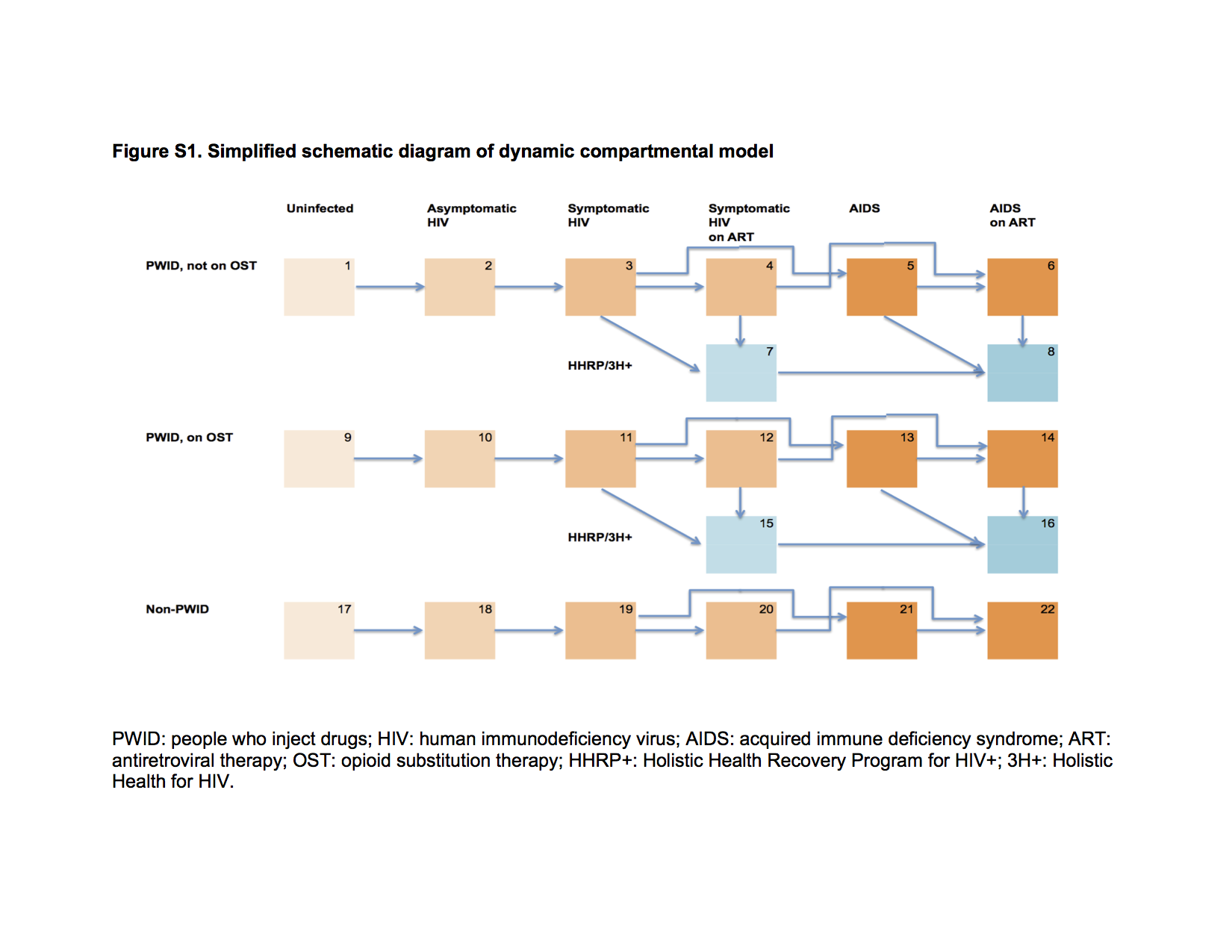

Supplement: S1 Fig — PWID: people who inject drugs; HIV: human immunodeficiency virus; AIDS: acquired immune deficiency syndrome; ART: antiretroviral therapy; OST: opioid substitution therapy; HHRP+: Holistic Health Recovery Program for HIV+; 3H+: Holistic Health for HIV. (TIFF) [file pone.0116694.s001.tiff]

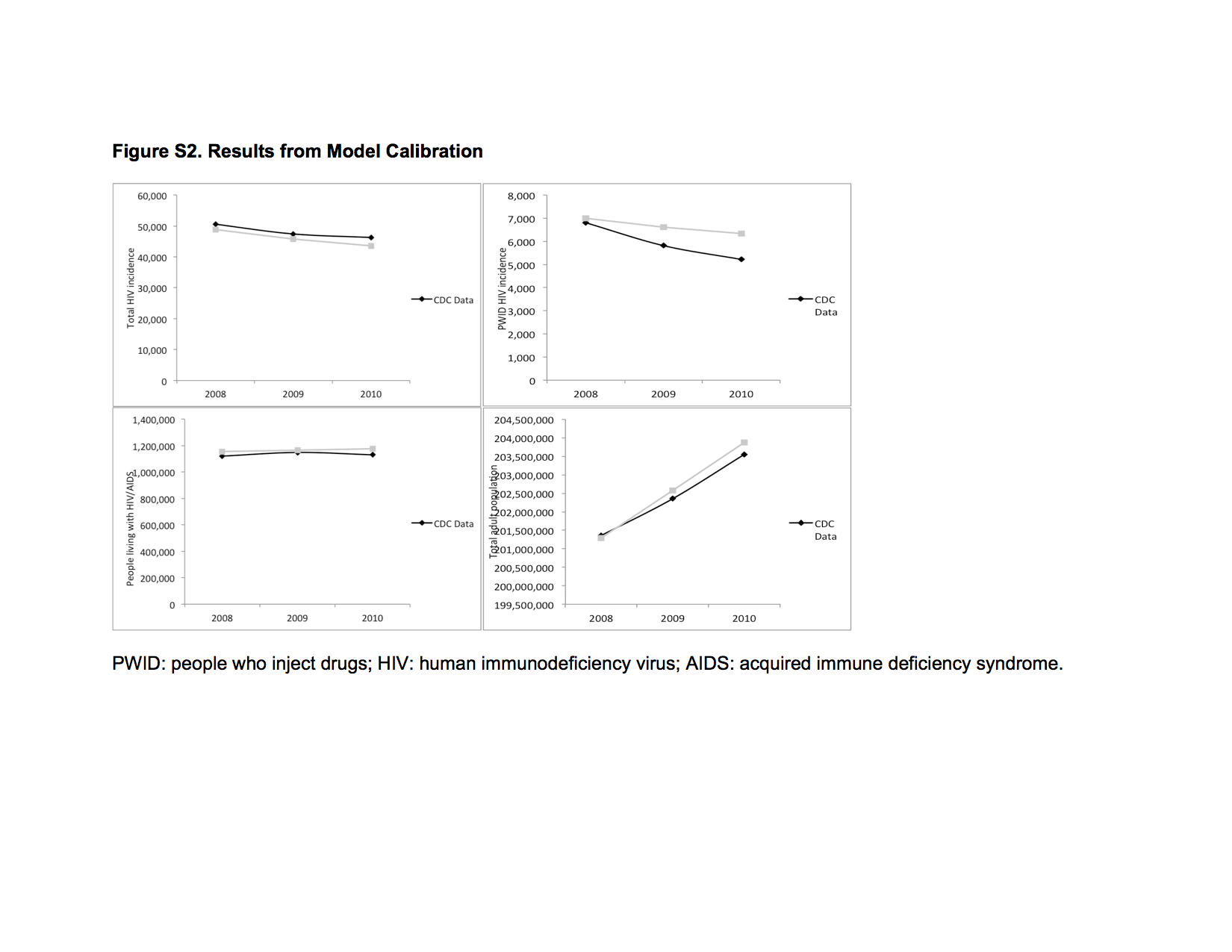

Supplement: S2 Fig — PWID: people who inject drugs; HIV: human immunodeficiency virus; AIDS: acquired immune deficiency syndrome. (TIFF) [file pone.0116694.s002.tiff]

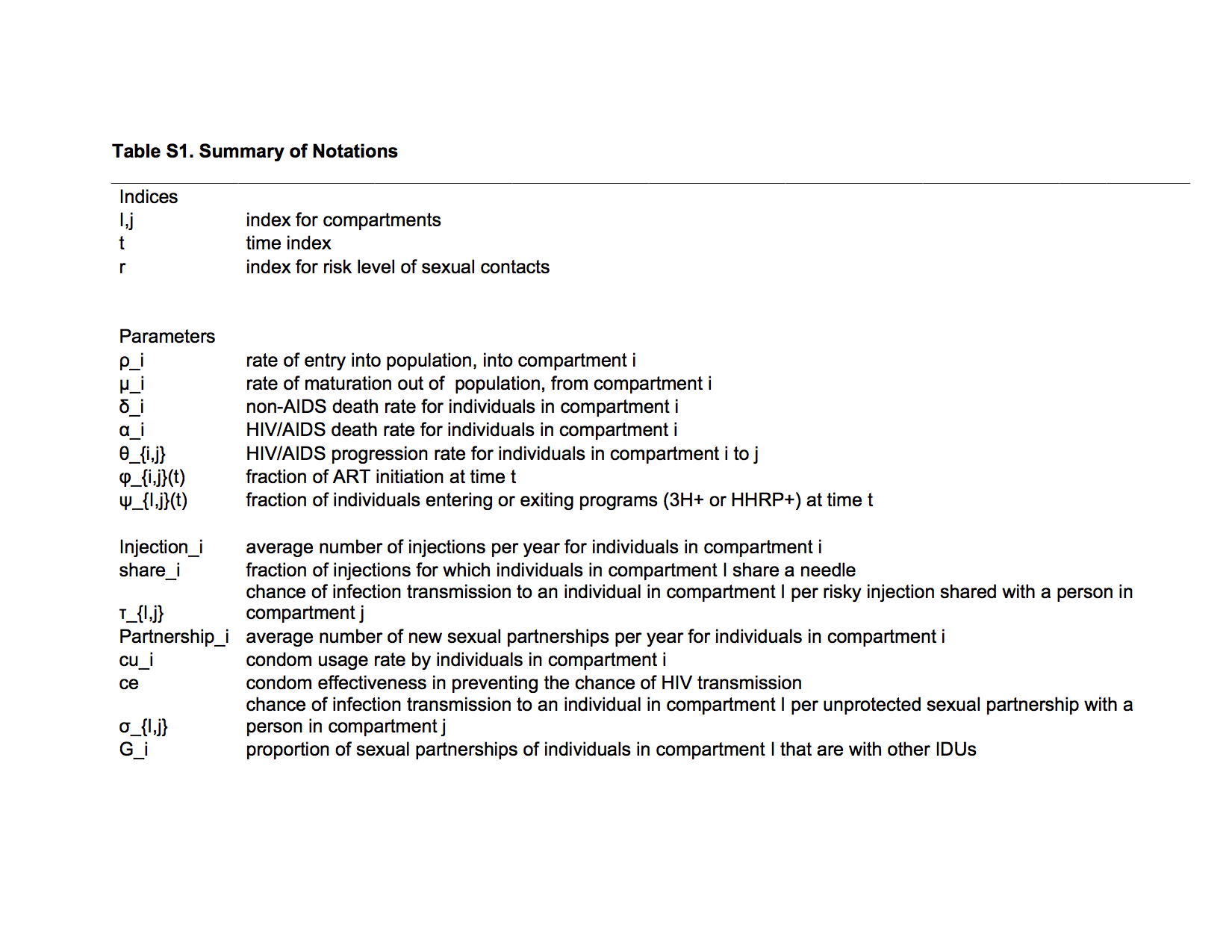

Supplement: S1 Table — HIV: human immunodeficiency virus; AIDS: acquired immune deficiency syndrome; ART: antiretroviral therapy; OST: opioid substitution therapy; HHRP+: Holistic Health Recovery Program for HIV+; 3H+: Holistic Health for HIV. (TIFF) [file pone.0116694.s003.tiff]

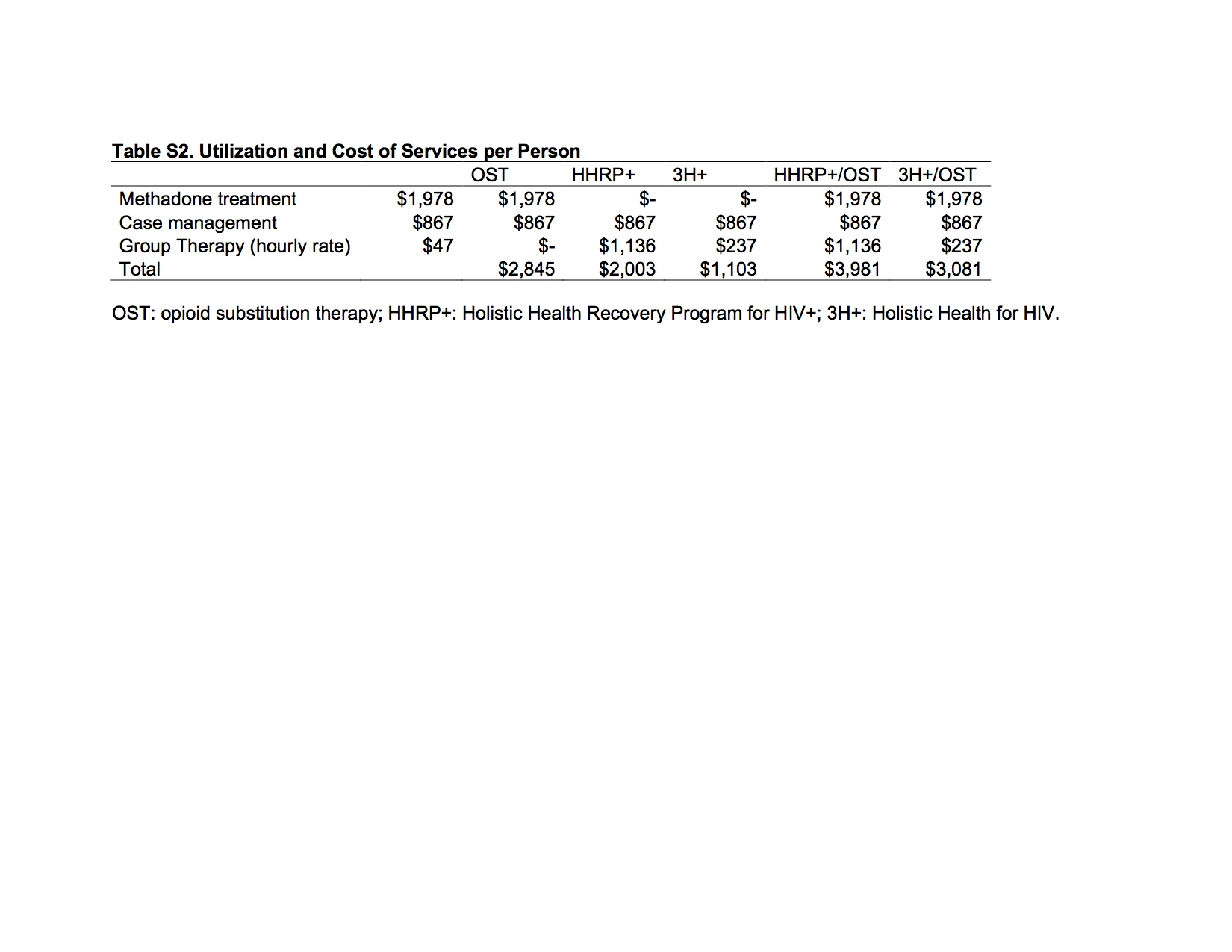

Supplement: S2 Table — OST: opioid substitution therapy; HHRP+: Holistic Health Recovery Program for HIV+; 3H+: Holistic Health for HIV. (TIFF) [file pone.0116694.s004.tiff]

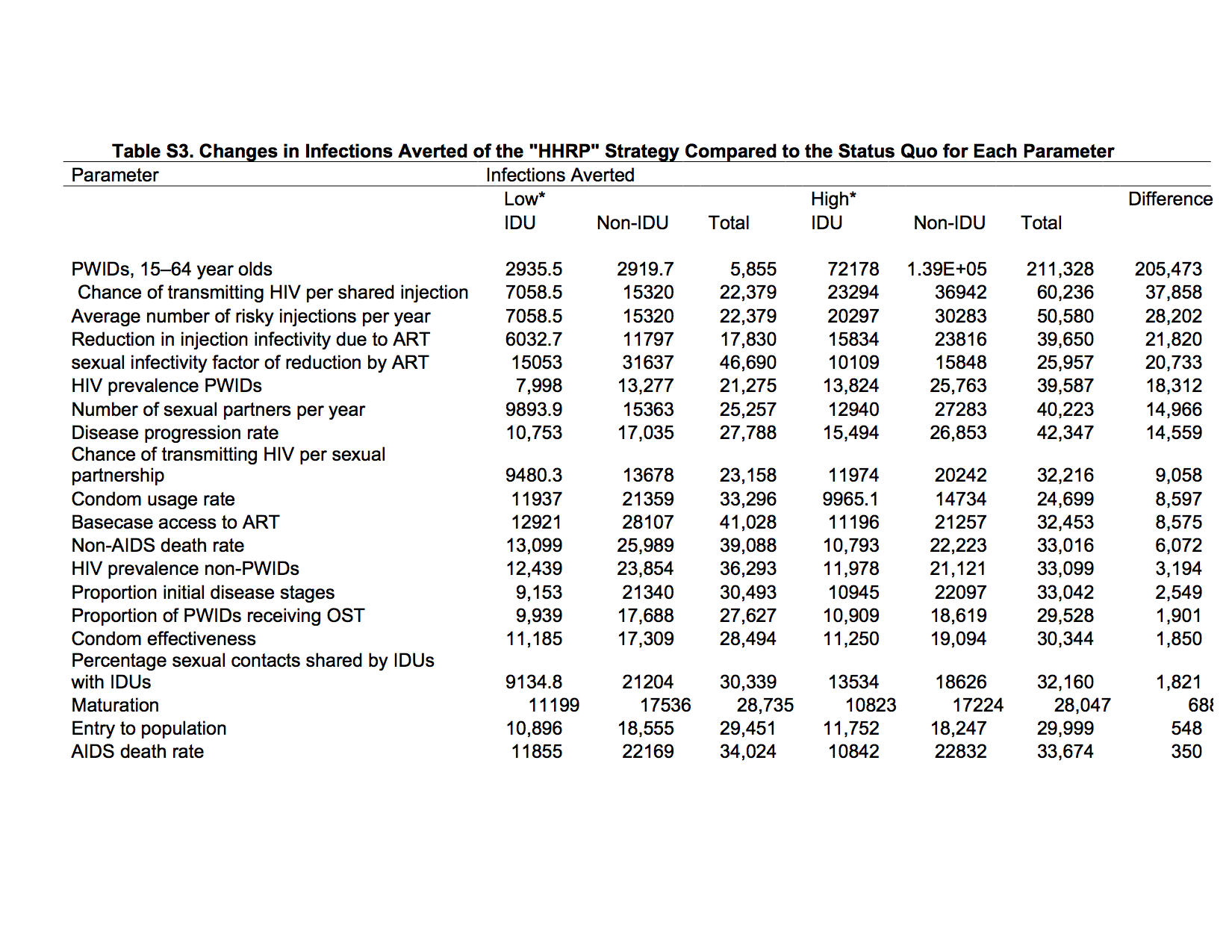

Supplement: S3 Table — PWID: people who inject drugs; HIV: human immunodeficiency virus; AIDS: acquired immune deficiency syndrome; ART: antiretroviral therapy; OST: opioid substitution therapy; HHRP+: Holistic Health Recovery Program for HIV+; 3H+: Holistic Health for HIV. (TIFF) [file pone.0116694.s005.tiff]

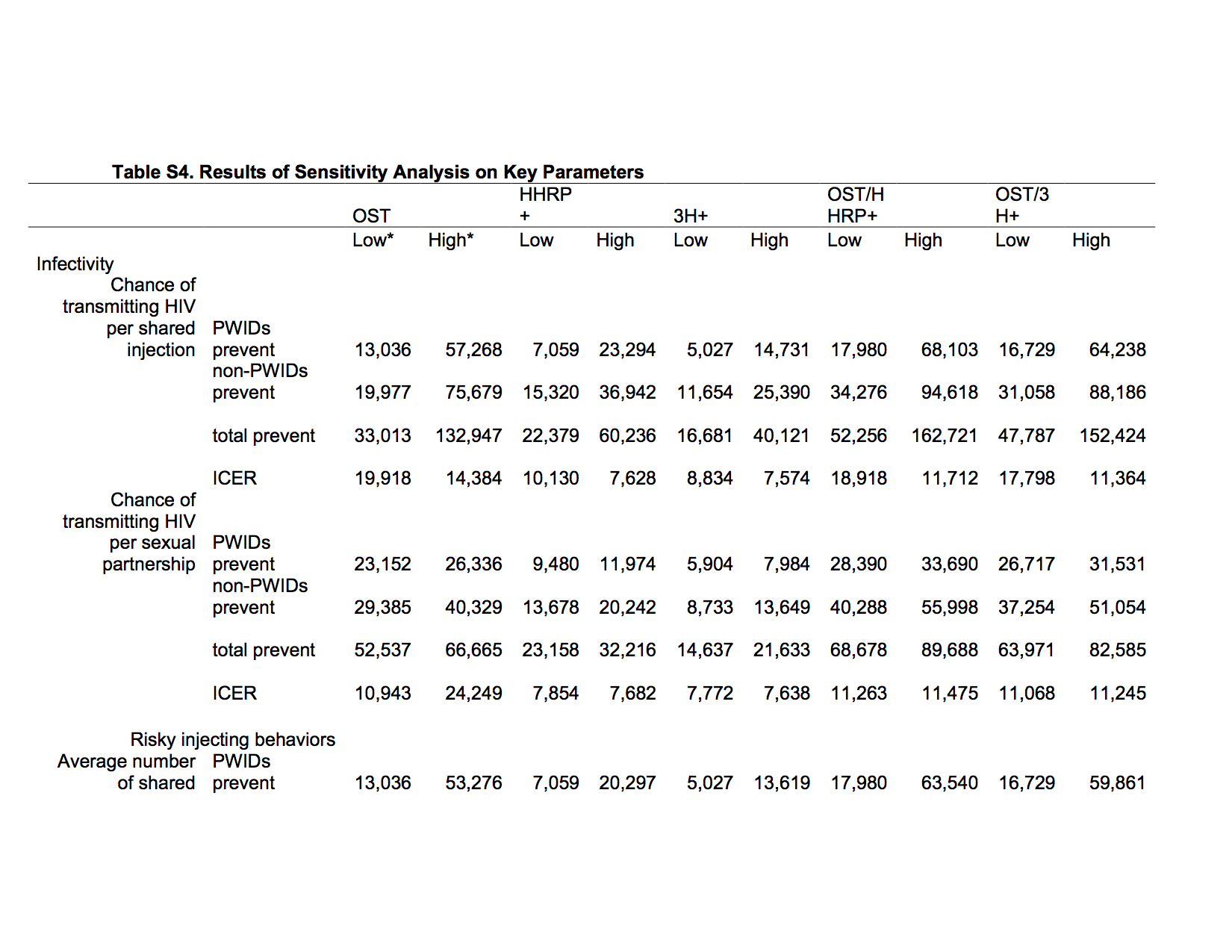

Supplement: S4 Table — PWID: people who inject drugs; HIV: human immunodeficiency virus; AIDS: acquired immune deficiency syndrome; ART: antiretroviral therapy; OST: opioid substitution therapy; HHRP+: Holistic Health Recovery Program for HIV+; 3H+: Holistic Health for HIV. (TIFF) [file pone.0116694.s006.tiff]
